# Bread and Roses: Social re‐presentations for Unconditional Basic Income in the Basque Country

**DOI:** 10.1111/bjso.12909

**Published:** 2025-06-09

**Authors:** Itziar Guerendiain‐Gabás, Maitane Arnoso‐Martínez, Lorena Gil de Montes

**Affiliations:** ^1^ Department of Social Psychology University of the Basque Country Donostia Spain; ^2^ ORAIN! Unconditional Basic Income Organization Donostia Basque Country Spain

**Keywords:** qualitative research, reflexive thematic analysis, social order, social representations, Unconditional Basic Income

## Abstract

The social debate on Unconditional Basic Income (UBI) has been growing in recent years. While the academic literature on social attitudes towards UBI has exploded, qualitative studies analysing the nuances and ideological conflicts that shape public debate remain scarce. Drawing on (action‐oriented) social representations theory and conceptions of social order, this study aims to delve into the shared meanings and arguments through which UBI is socially re‐presented, as well as the ideological objectives pursued by these arguments. To do so, we conducted a reflexive thematic analysis of 26 individual interviews conducted in the Basque Country. We generated four main themes: (1) Origins of social inequality; (2) The right to a good life; (3) Accessibility: Who should get a UBI; (4) Feasibility in the current system. Results indicated that the re‐presentation for UBI is anchored in socially disputed values of equality and freedom, which are objectified into conceptions of distributive justice and group stereotypes. This meaning‐making process seems to be conditioned by a social context marked by capitalist realism and political despair. We discuss these results from a theoretical and applied perspective, extending our understanding of how people collectively reason about UBI and what social‐psychological processes underpin it.

Universal Basic Income (UBI) is a policy proposal that has gained growing attention for guaranteeing income beyond employment in response to rising inequality and the increasing fragility of the labour market, particularly in the aftermath of the COVID‐19 pandemic (Johnson & Roberto, 2020). Considering the controversial and novel nature of this proposal, analysing the different beliefs, meanings, and arguments through which UBI is socially re‐presented seems particularly relevant, although this issue has been little addressed in the literature. Thus, we draw on (action‐oriented) social representations theory (SRT) (Buhagiar & Sammut, 2020) and Staerklé (2009)'s model of social order (SO) to study how the public argues for and against UBI. For this purpose, we conducted a reflexive thematic analysis (Braun & Clarke, 2019, 2021) on 26 individual interviews carried out in the Basque Country, a territory where the UBI debate has recently intensified. In doing so, we intend to broaden the theoretical understanding of the socio‐psychological processes by which UBI is collectively re‐presented, as well as to offer argumentative strategies to pro‐UBI social movements.

## Unconditional Basic Income

According to the Basic Income Earth Network (2024), UBI is defined as ‘a periodic cash payment unconditionally delivered to all on an individual basis, without means‐test or work requirement’. Thus, it is characterized by being: (1) individual, not dependent on the living unit; (2) universal, given to all persons regardless of their residency status; and (3) unconditional, without requirements or obligations of any kind (Raventós et al., 2017; Van Parijs & Vanderborght, 2017). Other relevant characteristics that are often discussed are sufficiency (i.e. its capacity to cover decent living standards) and redistribution (i.e. its capacity to generate top‐down fiscal redistribution); although these would depend on the specific UBI scheme proposed (e.g. financing mode, income amount, compatibility with pre‐existing social benefits). Traditionally, pro‐UBI arguments have revolved around (1) reducing poverty and ensuring decent living conditions for all; (2) overcoming the limitations of conditional minimum income schemes^1^; (3) providing greater bargaining power to workers in an increasingly precarious labour market; or (4) recognizing socially essential but unpaid activities (e.g. care work). In contrast, typical arguments against UBI are related to (1) the impossibility of financing it; (2) reducing the incentive to work and encouraging laziness; or (3) causing a pull effect for immigration (for an overview, see Afscharian et al., 2022).

Academic literature has predominantly focused on the philosophical debate and economic feasibility of UBI, while interest in its psychological feasibility—that is, its ability to mobilize positive emotions and arguments among the public (De Wispelaere & Noguera, 2012)—has been recent. Several survey studies have examined public opinion on UBI, showing that support varies across socio‐political contexts but tends to be stronger in countries with higher inequality and weaker welfare systems (e.g. Lee, 2018; Parolin & Siöland, 2020; Roosma & van Oorschot, 2020; Yang et al., 2024). In addition, research has found that support for UBI is higher among groups with lower socio‐economic status (i.e. lower income groups, the unemployed, etc.) (e.g. Guerendiain‐Gabás et al., 2024; Kim et al., 2025; Roosma & van Oorschot, 2020; Vlandas, 2021; Yang et al., 2024); consistent with a group‐based motivation to reduce uncertainty and strive for greater (economic) security (Hasenfeld & Rafferty, 1989). Complementarily, research has proved that support for UBI is stronger among leftists (e.g. Parolin & Siöland, 2020; Roosma & van Oorschot, 2020; Vlandas, 2021; Yang et al., 2024) and those who hold egalitarian and redistributive values (e.g. Lee, 2021; Patulny & Spies‐Butcher, 2023; Roosma & van Oorschot, 2020; Sureth et al., 2024). Conversely, system‐legitimizing beliefs (e.g. meritocratic values, individualistic attributions of poverty) and welfare chauvinism predict lower support for UBI (e.g. Baranowski & Jabkowski, 2021; Lim & Tanaka, 2019; Parolin & Siöland, 2020; Roosma & van Oorschot, 2020).

Nevertheless, previous studies remain somewhat limited, as assessing support for UBI using single‐item measures fails to capture the complexity of how people understand this proposal or what their arguments are for and against it (Roosma & van Oorschot, 2020). To fill this gap, recent studies have analysed the multidimensionality of attitudes towards UBI, comparing levels of support for different types of basic income (e.g. Kim et al., 2025; Laenen et al., 2023; Rincón, 2023, 2024). Meanwhile, qualitative research capable of exploring these issues more comprehensively has been rather scarce. To our knowledge, few qualitative studies have been conducted, mostly in Europe (Herke & Vicsek, 2022; Rossetti et al., 2020; Zimmermann et al., 2020)—with some exceptions in Latin America (Acuña Gómez et al., 2023). These studies show that debates on UBI are rooted in values of equality and freedom, and generally revolve around the (un)conditionality of the scheme (who should be entitled to receive this income) and its political and economic feasibility (how possible it is to implement a UBI in practice). However, this research has been predominantly descriptive in nature, often neglecting a broader theoretical analysis of the psychosocial processes underlying the formation of attitudes towards UBI. Thus, the present study aims to advance research in this sense. Drawing on (action‐oriented) SRT and normative conceptions of SO, we seek to generate a comprehensive theoretical framework on how shared meanings and beliefs about UBI are generated and used pragmatically to move this political project forward (vs. backward).

## (Action‐oriented) social representations theory and conceptions of social order

Social representations are culturally elaborated systems of meanings and beliefs that structure our understanding of the social world (Moscovici, 1961). They are concerned with making the unfamiliar familiar, constituting common sense knowledge that guides social attitudes, ideologies, and practices (Sammut et al., 2015). This occurs mainly through two psychosocial processes: anchoring, whereby new objects and meanings are integrated into previous knowledge, and objectification, whereby abstract ideas are made concrete by making an image or metaphor correspond to an object (Rateau et al., 2011). Importantly, social representations are actively constructed and negotiated within the social space, shaped by inter‐ and intra‐group communication and power relations (Staerklé, 2009). In this sense, they are a source of argumentation, dilemmatic thinking, and resistance; fulfilling specific ideological functions: common sense can serve both to support and to resist the status quo (Howarth, 2006).

From this approach, policy attitudes are seen as symbolic tools with which individuals and groups assess the desirability and legitimacy of different models of society (Staerklé, 2009; Staerklé et al., 2012). As part of political lay thinking, policy attitudes are anchored in normative beliefs, that is, widely disseminated ideas and shared understandings regarding social organization and the just distribution of resources. These normative beliefs function as the organizing principles of attitudes, crystallizing in competing definitions of social order which prescribe different types of relationships between individuals, groups and institutions (Staerklé, 2009; Staerklé et al., 2012). These conceptions of SO are objectified through stereotypes of value‐conforming and value‐violating groups, transforming abstract ideological values into socially useful knowledge about the perceived entitlement of potential beneficiary groups; thus guiding positioning towards a given social policy. Specifically, Staerklé (2009)'s model distinguishes four conceptions of SO, resulting from the intersection of two fundamental polarities: (1) normative (within‐group) versus categorical (between‐group) differentiation, (2) defined by identity (symbolic) versus positional (material) concerns (see Table 1). Nevertheless, these four conceptions are not mutually exclusive, but rather blend and combine in multiple ways; giving rise to the complexity of political lay thinking (Staerklé, 2009; Staerklé et al., 2012).

**TABLE 1:** Model of lay conceptions of social order (Staerklé et al., 2012).

	Social identity	Social position
	Moral order	Free market
Normative differentiation		
Principle of categorisation	Morality	Productivity
Core antagonisms	Good vs. bad	Winners vs. losers
Principle of social regulation	Conformity; similarity	Equity; self‐interest
Welfare policies	Private support; charity	Private responsibility; insurance
Normative beliefs	Authoritarianism; distrust	Welfare dependency

	Social diversity	Structural inequalities
Categorical differentiation		
Principle of categorisation	Social heterogeneity	Social class, status
Core antagonisms	Majority vs. minority	Dominants vs. subordinates
Principle of social regulation	Inter‐group differentiation	Inequality management
Welfare policies	Majority favouritism vs. Group rights	Elite favouritism vs. Redistribution
Normative beliefs	Ethnocentrism vs. Multiculturalism	Social dominance vs. Egalitarianism

*Note*: From Staerklé et al. (2012).

First, the conception of Moral order arises from symbolic, ingroup differentiation and focuses on conformity to social norms, establishing a normative divide between ‘good’ and ‘bad’ citizens based on their adherence to or deviation from core societal values. The Free market conception, in turn, stems from ingroup differentiation but in positional terms: values of individualism and meritocratic achievement emphasize competitive relations between ‘winners’ and ‘losers’, re‐presenting ‘free‐riders’ (i.e. those who do not work) as a threat to social order. In contrast, Social diversity is based on categorical distinctions between groups (ingroup vs. outgroup) defined by essentialised traits. This conception is more complex, as group differences can be evaluated as either positive, for example, in movements defending minority rights, or negative, for example, in discriminatory thinking and behaviour. Finally, in the conception of Structural inequalities, lay political thinking is structured by group hierarchy and class‐based differences (dominants vs. subordinates). Again, this conception is complex in that structural inequalities can be perceived either as legitimate, reinforcing social hierarchy, or as illegitimate, promoting inequality reduction and system change.

Consequently, conceptions of SO shape and legitimize policy attitudes, thus pursuing transformative versus conservative political ends (Staerklé, 2009; Staerklé et al., 2012). This notion is in line with recent SRT approaches that emphasize the action implicit in social representations (see Buhagiar & Sammut, 2020). Action‐oriented approaches conceptualize knowledge construction as an active social re‐presentation in the context of an inter‐group conflict, focusing on the re‐presentation goal of going for or against a specific joint project (Buhagiar & Sammut, 2020, 2023). This theoretical framework brings social representations closer to discursive perspectives (e.g. Billig, 1987), underscoring the pragmatism and ideological functionality of discourse (Castro & Batel, 2008). This conceptualization is particularly relevant for studying the social re‐presentations of UBI, as it is a new and highly contested policy in the public sphere, which entails a discursive struggle between conflicting social groups to position it as a desirable (vs. detrimental) proposal. Thus, analysing social re‐presentations for UBI from this approach allows us to pay attention both to (1) the normative beliefs that shape shared meanings and arguments and (2) to the pragmatic ends pursued by these arguments.

## Study context

This research was carried out in the Basque Country, a context where the UBI debate has been ongoing for several decades. Despite having one of the most advanced conditional minimum income schemes in Spain, various social organizations have for years been criticizing its insufficiency and exclusionary nature, calling for a UBI instead (e.g. Ezker Sindikalaren Konbergentzia, 2016). The COVID‐19 pandemic reignited public debate on UBI by exposing the limits of the existing welfare model and highlighting the need for income beyond employment. This led to the organization of a civic campaign to take the proposal for UBI to the Basque Parliament. The campaign gathered over 20,000 signatures and 80 endorsements from social movements, consequently bringing the proposal to Parliament. Although it was finally rejected—only left‐wing parties Elkarrekin Podemos‐IU and EH Bildu voted in favour—the initiative launched an ongoing political campaign that raised public awareness and amplified pro‐UBI arguments (see Guerendiain‐Gabás & Arnoso‐Martínez, 2022 for a summary of this process).

## Study aims

This study intertwines an action‐oriented SR approach (Buhagiar & Sammut, 2020) and Staerklé (2009)'s model of SO to examine the following research questions: (1) How is the UBI proposal socially re‐presented? (process) and (2) What are these re‐presentations used for? (ends). Specifically, we aim to analyse (1) how shared meanings about UBI are socially constructed through anchoring and objectification into different conceptions of SO and normative beliefs and (2) how these are used to articulate arguments to pursue different ideological objectives and move the joint project of UBI forward versus backward. Ultimately, we seek to deepen the theoretical understanding on how social re‐presentations about UBI are generated and to inform pro‐UBI social movements on how to strengthen social support for this proposal.

## METHOD

### Participants

Heterogeneous purposive sampling (Douglas, 2022) was employed to achieve maximum variation in discourse according to different socio‐demographic characteristics (gender, age, employment status, socio‐economic status and political stance). Participants were recruited through informational flyers and snowballing until saturation and sufficient heterogeneity were reached. Specifically, 26 people were interviewed, all over the age of 18 and living in the Basque Country. Participants' main socio‐demographic characteristics are summarized in Table 2. For complete socio‐demographic characteristics of each study participant, see Table S1.

**TABLE 2:** Socio‐demographic characteristics of study participants.

	n	%
Gender		
Woman	10	38.5
Man	14	53.8
Non‐binary	2	7.7
Age		
18–24 years	2	7.7
25–34 years	8	30.8
35–44 years	5	19.2
45–54 years	3	11.5
55–64 years	8	30.8
Employment status		
Employed	20	76.9
Unemployed	1	3.8
Student	2	7.7
Retired	2	7.7
Household and care work (unpaid)	1	3.8
Social class		
Lower/lower‐middle class	9	34.6
Middle class	8	30.8
Upper/middle‐upper class	9	34.6
Political orientation		
Left	16	61.5
Center	4	15.4
Right	6	23.1
Level of information on UBI		
Low	14	53.8
Medium	9	34.6
High	3	11.5
Stance on UBI		
In favour	15	57.7
Against	8	30.8
Unclear	3	11.5

### Procedure

Data were collected in Spanish through semi‐structured individual interviews, between September and December 2023. Participants signed an informed consent form before the interview began. Participation was voluntary, with no compensation of any kind. Interviews lasted between 41 and 93 min (average duration: 61 min). The interviews were audio‐recorded and transcribed afterwards. The script of the interview was as follows: (1) socio‐demographic characteristics; (2) definition of UBI^2^ (explicitly mentioning its individuality, universality, unconditionality, sufficiency and redistributive capacity); (3) open‐ended questions on participants' opinions around UBI, supplemented with why questions (Flick et al., 2015); (4) final conclusions, previous level of information, and explicit stance for or against UBI. The full interview script can be found in the Table S2.

### Analytic framework

A reflexive thematic analysis (Braun & Clarke, 2019, 2021) was conducted using ATLAS.ti software. As Braun and Clarke (2021, p. 11) argue, reflexive thematic analysis allows us to investigate ‘the social or discursive construction of particular ‘social objects’, subject positions, or other social phenomena in particular contexts and the implications and effects of these’; hence we consider it appropriate to address our research question. We adopted a mainly inductive and theoretically sensitive approach to analysis, underpinned by a ‘weak’ socio‐constructivist epistemological perspective (Jovchelovitch, 2007). Themes were developed from the participants' narratives (the main categories were not predetermined) and were re‐elaborated during the analysis on the basis of relevant scientific literature on UBI, SRT and political psychology, as well as the authors' own activist experience in pro‐UBI social movements. From the perspective of situated knowledge (Haraway, 2013) and reflexivity (Braun & Clarke, 2019, 2021), we understand analysts' subjectivity not as an obstacle that taints the veracity of scientific knowledge, but as a valid and useful resource for analysis.

The analysis began by re‐reading the transcribed interviews and taking notes. Following the research objective, the analysis aimed to explore the discourses and arguments used by the participants when debating UBI. An initial coding of the discourses/arguments generated by each participant was carried out by the first author and subsequently discussed among all authors. Following Braun and Clarke (2019, 2021), this discussion was not intended to seek objective agreement among the analysts (e.g. through inter‐judge reliability assessments), but rather to develop as rich and nuanced an analysis as possible. The identified discourses/arguments were then re‐grouped into new codes (considering all interviews globally) and an inductive thematic categorization was carried out to group the codes by broader patterns of meaning to form potential themes. The suitability of each candidate theme was reviewed against the coded data and the entire dataset to determine whether it told a compelling story and answered our research question. This way, the contents of each theme were specifically analysed, taking into account how social re‐presentations about UBI were generated and the pragmatic ends pursued by these re‐presentations. Candidate themes were further developed by constructing superordinate themes to create more abstract stories about the data (main themes), while creating sub‐categories to make explicit the nuances within each core story (sub‐themes). This process was carried out in dialogue with previous literature and researchers' own experiences. Candidate themes were continuously debated and re‐elaborated until a consensus was reached among all authors.^3^

## RESULTS

**FIGURE 1:**
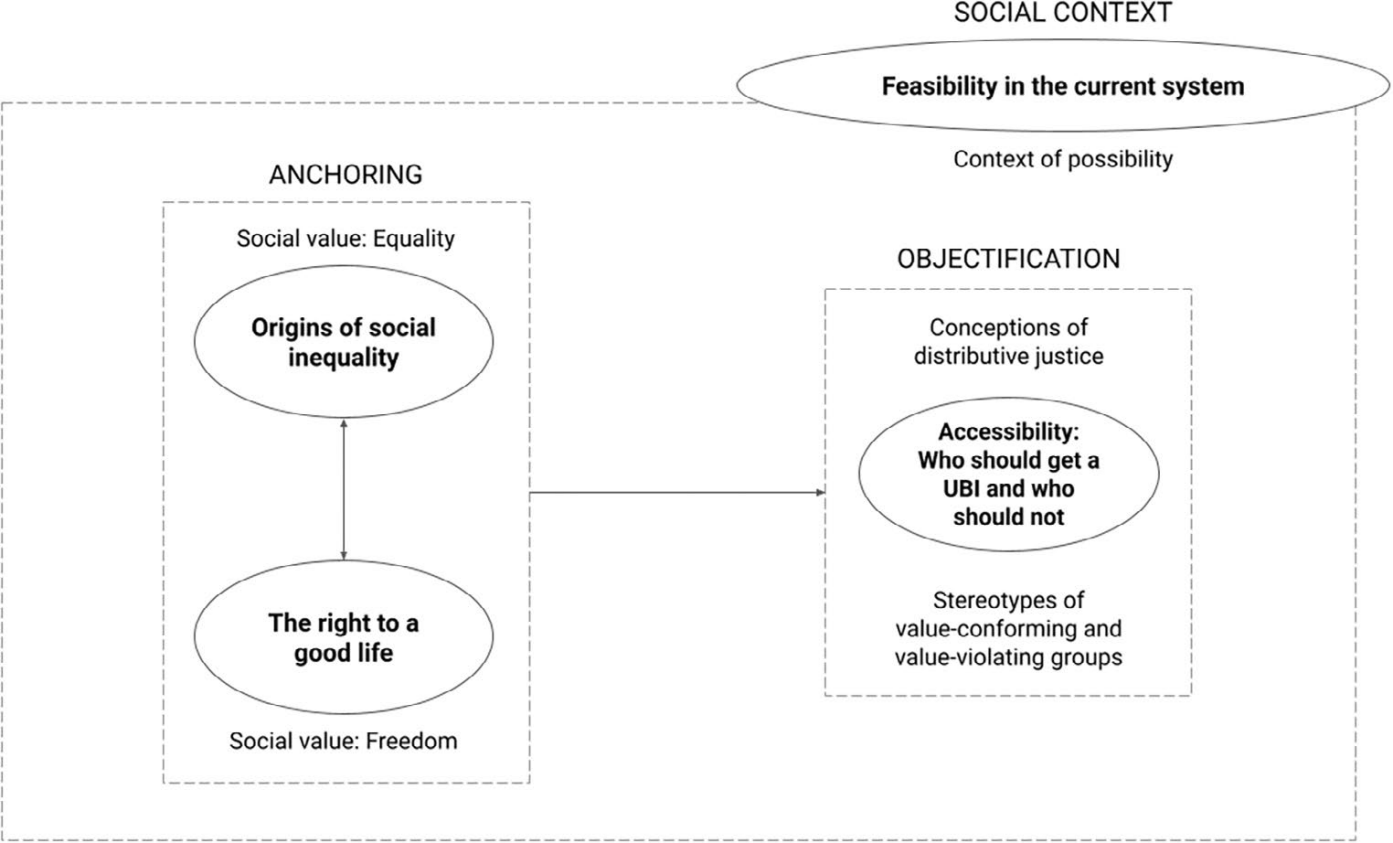
Thematic structure generated from reflexive thematic analysis.

We generated four main themes to explain how people re‐present UBI: (1) Origins of social inequality; (2) The right to a good life; (3) Accessibility: Who should get a UBI and who should not; and (4) Feasibility in the current system. Themes 1 and 2 refer to the ideological values and normative beliefs on which the discussion around UBI is socially anchored; Themes 3 capture the way in which re‐presentations for UBI are objectified around conceptions of distributive justice and group stereotypes; and Theme 4 concerns the social context in which this re‐presentation takes place, that is, the context of possibility to implement a UBI (see Figure 1 and Table 3). Each theme is divided into its respective sub‐themes, and sample quotations are provided to support our analysis. All quotations have been translated into English for the writing of this article, but the Spanish originals, as well as supplementary quotations, can be found in the Table S3. Other supplementary material (e.g. full interview transcripts, coded files, etc.) is not publicly available due to privacy issues, but can be requested from the corresponding author.

**TABLE 3:** Theoretical model generated from reflexive thematic analysis.

Theme/sub‐theme	Psychosocial processes	Social order
Anchoring		
1. Origins of social inequality	Contested social value: Equality	
1.1. Inequality as an individual issue “Those who earn a lot, it's because they work a lot”	Individual attributions of inequality System justification	Free market
1.2. Inequality as a structural issue “There are poor people because there are very rich people”	Structural attributions of inequality System questioning	Structural inequalities
2. The right to a good life	Contested social value: Freedom	
2.1. Freedom as a collective issue “We would feel freer to choose”	Collective aspirations for freedom System questioning	Structural inequalities
2.2. Freedom as an individual issue “I would like to be financially independent”	Individual aspirations for freedom System justification	Free market
Objectification		
3. Accessibility: Who should get a UBI and who should not	Conceptions of distributive justice Stereotypes of (un)deserving groups	
3.1. Need‐based distribution “A basic income for those who need it”	Distribution criterion: need Stereotypes: the rich parasite; the good citizen	Structural inequalities;Moral order
3.2. Reciprocity‐based distribution “If you want to receive, you have to give something in return”	Distribution criterion: equity Stereotype: the parasite	Free market
3.3. Autochthony‐based distribution “UBI would have a pull effect”	Distribution criterion: autochthony Stereotype: the racialized other	Social diversity
Deservingness considerations challenged by criterion of equality (human rights framework)		
Social context		
4. Feasibility in the current system	Representations of the future (feasibility)	
4.1. Unfeasibility “UBI is impossible to implement”	Dystopian future; capitalist realism	Moral order
4.2. Towards utopia “UBI is a challenge to capitalism”	Utopian future; social change	–

### Theme 1. Origins of social inequality

This theme captures how arguments about UBI are anchored in normative beliefs about current social organization and the roots of inequality. Specifically, re‐presentations for UBI are underpinned by the tension between individualistic versus structural explanations of social inequality (Furnham, 1982; Kluegel & Smith, 2017), which serve to legitimize versus challenge the status quo (Jost, 2019; Jost & Banaji, 1994) and guide participants' position towards UBI. This tension reflects the interplay between the free market and structural inequalities conceptions of SO, since social hierarchy and beliefs about the (un)desirability of inequality frame political lay thinking (Staerklé, 2009; Staerklé et al., 2012).

#### Sub‐theme 1.1. Inequality as an individual issue: Those who earn a lot, it's because they work a lot

The re‐presentation of inequality as an individual issue (Furnham, 1982; Kluegel & Smith, 2017) is anchored on the naturalization and justification of class differences through system‐legitimizing myths (Jost, 2019; Jost & Banaji, 1994). Following a free market conception of SO (Staerklé, 2009; Staerklé et al., 2012), discourses of meritocracy, self‐sufficiency, and work ethic are used to hold individuals responsible for their poverty situation and represent wealth as the result of hard work and effort (Bauman, 1999; Weeks, 2011). This provides a rationale for the rejection of inequality‐reducing, redistributive social policies such as UBI (Sidanius et al., 2016; Sidanius & Pratto, 1993).

I don't think anyone is given free money: those who earn a lot, it's because they work a lot. Maybe there is a 1%, the football player, the lucky one, Amancio Ortega…^4^ but of the remaining 99%, those who earn a lot, it is because it takes a lot of time and effort. (P10)

Nevertheless, some exceptions are recognized where wealth may be the result of luck rather than effort, or where state support for the poor may be justified (e.g. in situations of illness, homelessness, etc.); reflecting the difficulty of reconciling conflicting cultural values such as egalitarianism and the work ethic (Staerklé, 2009). This ambivalence is resolved by individualizing poverty intervention in the form of an aid (as opposed to a right), which implies intervention that is targeted (only for deserving groups), minimal (sufficient to survive, but not so high as to lift people out of poverty) and temporary (aimed at getting beneficiaries back to work).

Take away the subsidies. If you are fit to work, you work. There is no aid. Either that, or the differential should be much higher; basic income should be a minimum allowance, so that you can eat a bowl of soup every day and that's it. (P13)

#### Sub‐theme 1.2. Inequality as a structural issue: There are poor people because there are very rich people

In contrast, an alternative re‐presentation of inequality as a structural issue is constructed to contest the previous narrative (Furnham, 1982; Kluegel & Smith, 2017). Based on a Structural inequalities conception of SO, power differentials between the rich and the poor are deemed fabricated and illegitimate; thereby objectifying poverty as a crime of the capitalist system and explaining wealth as the result of the illegitimate hoarding of resources (Marx, 1908): ‘There are poor people because there are very rich people’ (P24). This denaturalisation of class differences creates a re‐presentation of inequality in terms of group injustice and shared grievance, that motivates mobilization to change the status quo (van Stekelenburg & Klandermans, 2013).

I think poverty is a crime: to be born in Ondarreta or in Altza,^5^ and for the difference in mortality to be eight years, I think it's a fucking crime. (P6)

This structural re‐presentation facilitates pro‐UBI arguments by embedding them in shared values of egalitarianism and collective responsibility (Patulny & Spies‐Butcher, 2023; Roosma & van Oorschot, 2020), as well as in the idea of the right to existence (Casassas, 2018; Raventós, 2021): in a society where the satisfaction of basic needs (food, housing, etc.) depends on income, this should be guaranteed for everyone by the mere fact of being alive. As opposed to the representation of UBI as an aid, this allows participants to identify UBI as a social right that must be guaranteed by the institutions—just like other welfare rights that enjoy greater social consensus (e.g. health, education); thus legitimizing the making of this claim to the state.

Everyone has the right to live with dignity. And to live with dignity in this world, we need money. Because that's how it works. So I think it's fair. We all deserve that just because we live in this shitty society. (P7)

### Theme 2. The right to a good life

Alongside (in)equality and the just organization of resources, social re‐presentations about UBI also revolve around the satisfaction of post‐materialistic needs (e.g. well‐being, emancipation, social participation) (Inglehart & Welzel, 2010). This discussion is anchored in the shared value of freedom and normative beliefs about what constitutes a good life, a life that is worth the joy of being lived (Fulladosa‐Leal et al., 2021; Pérez‐Orozco, 2019). Nevertheless, these desires for freedom are complex re‐presentations as they can be individualised—reinforcing social hierarchy—or collectivised—striving for collective emancipation (Jost, 2019; Jost & Banaji, 1994); thus reflecting again the tension between the free market and structural inequalities conceptions of SO (Staerklé, 2009; Staerklé et al., 2012).

#### Sub‐theme 2.1. Freedom as a collective issue: We would feel freer to choose

Advancing the Structural inequalities conception of SO (Staerklé, 2009), participants anchor pro‐UBI arguments not only in the promotion of equality of resources between groups, but also in demands for social emancipation and a livable life for the subordinate classes—reminiscent of the historical slogan of the striking women: ‘Bread for all, and Roses, too’. These claims are anchored in values of collective freedom, following the republican approach of freedom as non‐domination (Casassas, 2018; Raventós, 2021). This conception is based on a critique of paid work as an obligation that capitalism imposes on us in order to survive, and the consequences this has in terms of actual freedom of choice for subordinate groups (Frayne, 2015; Weeks, 2011). Within this framework, UBI is constructed as a policy that would increase our autonomy, allowing us to make life decisions without being subject to wage dependence (Gorz, 1999).

In this village, since we were 14 years old, we have all had to work. And there were no other options. So, with basic income all that would end, wouldn't it? We would feel freer to choose. (P22)

In this way, categorical comparisons between the working class and the ruling classes are made to advance emancipatory demands; but also with other historically subordinated groups such as women (vs. men), following the demands for UBI as a policy beyond the work‐and‐family system (Weeks, 2011): ‘Now it's a dependency on my husband, otherwise it would be a dependency on my company… I envision more independence to be able to choose, right?’ (P9). Overall, the lay understanding of a good life is objectified around images of leading a slower pace of life, achieving peace of mind by reducing economic worries, and improving well‐being and community cohesion; thereby creating a shared imagined future that helps to drive the joint project of UBI forward (Buhagiar & Sammut, 2023).

It would take away the pressure that a lot of people have of not making ends meet, because you have a shit salary, and it doesn't allow you to have a normal life. If you take that part out, people would live a much better life. (P1)

#### Sub‐theme 2.2. Freedom as an individual issue: I would like to be financially independent

Living without working, however, is a more generalized aspiration not exclusive to those who question capitalism and support UBI as a tool for collective emancipation. The quest for an autonomous life is also anchored in a neoliberal and individualized conception of freedom, which ignores the social circumstances and structural inequalities that condition people's lives (fantasy of individuality; Hernando, 2018). Thus, this discourse emphasizes individual effort and astuteness as means to emancipate oneself from work (e.g. through private investments that allow the accumulation of wealth), while rejecting the public provision of a UBI that would collectivize this emancipation, as this would encourage free‐riding and social parasitism (Likki & Staerkle, 2014). This re‐presentation is rooted in a free market conception of SO, where social relations are regulated on the basis of individual competition (Staerklé, 2009; Staerklé et al., 2012). In this way, the right to a good life is individualized as a privilege reserved only for the ‘winners’ of society—and denied to the ‘losers’; legitimizing the status quo by subscribing to the hegemonic myths of meritocracy and self‐sufficiency (Jost, 2019; Jost & Banaji, 1994).

I would like to be financially independent, and be able to live without working; but not living by taking advantage of others. Instead, I want to collect rents or invest money so that I can get there. (P16)

### Theme 3. Accessibility: Who should get a UBI and who should not

Social re‐presentations for UBI are objectified in participants' discourse around normative beliefs about distributive justice (Veghte, 2016) and deservingness considerations (van Oorschot, 2000), crystallizing into group stereotypes (Staerklé, 2009; Staerklé et al., 2012). In this way, stereotypes provide an important link between abstract ideological values (i.e. equality, freedom) and policy attitudes (Staerklé, 2009), making the stance towards UBI concrete by creating images of (un)deserving recipient groups. Specifically, participants objectified re‐presentations using three distributive criteria: need, reciprocity, and autochthony; each of which conflicts in its own way with an egalitarian redistribution of resources.

#### Sub‐theme 3.1. Need‐based distribution: A basic income for those who need it

Under the need criterion (van Oorschot, 2000), participants argue that social assistance should be targeted at those who truly need it. Based on a Structural inequalities conception of SO, a categorical differentiation is constructed between rich and poor people, which is objectified in the stereotype of the ‘rich parasite’ who takes advantage of the lower and middle classes (Staerklé, 2009; Staerklé et al., 2012): ‘Why on earth would a rich person want to receive 900 euros… but help the person who earns 1,200 euros, right?’ (P10). Once the scope of redistribution is narrowed to the ingroup (i.e. subordinate classes), intra‐group subcategorisation (e.g. according to household size, specific situations of vulnerability such as illness, etc.) leads to the elaboration of differentiating deservingness judgements in terms of (un)conformity to ingroup values, such as the expectation that welfare recipients behave as good citizens—thus adopting a Moral order conception of SO (Staerklé, 2009; Staerklé et al., 2012). This provides a rationale for determining who is (un)deserving of UBI and legitimizes the preference for a needs‐based distribution of resources (see Wenzel, 2004), incompatible with the universal nature of UBI.

A couple with one child is not the same as a couple with four children… But I'm talking about situations of vulnerability, when one is ill, when the mother of another comes to look after him… Helping people on a case‐by‐case basis. But an uncontrolled distribution, for everyone? No, I disagree. (P3)

Conversely, in favour of universality, the problems of conditional minimum income schemes are identified (e.g. complex bureaucratic procedures); arguing that a UBI for all, including the rich, would guarantee access to this income to those who are usually excluded from social assistance, avoiding the social stigmatization of beneficiaries (Casassas, 2018; Raventós, 2021). In this case, neediness is constructed as a deviation from a state that should apply equally to all (i.e. access to decent living conditions), so the appeal to a need‐based criterion of justice leads to a non‐differentiating distribution in egalitarian terms (Wenzel, 2004). This is made possible by overcoming intra‐group subcategorisation and adopting an inclusive group identity, based on a broader reference category (e.g. we, everyone, etc.), which serves to objectify UBI as a human right and confront moral considerations about who is a good welfare recipient.

If we all received it, things would change. When only a few receive it, they are discriminated against. Because the rest have to get it with sacrifice. But if they give it to everyone, it would be easier. (P22)

#### Sub‐theme 3.2. Reciprocity‐based distribution: If you want to receive, you have to give something in return

Regarding the reciprocity criterion (van Oorschot, 2000), social contribution is emphasized as a deserving condition to access common resources: ‘Everyone has rights and obligations in this society. OK, you have the right to get this, but… What good are you going to do for society?’ (P16). This narrative is underpinned by the neoliberal rhetoric that ‘there can be no rights without responsibilities’ (Dean, 2004), which is anchored in an economic conception of citizenship that defines social contribution as participation in the labour market (Crespo‐Suárez & Serrano‐Pascual, 2016; Serrano‐Pascual et al., 2012). Following a Free market conception of SO, reciprocity‐based conceptions are objectified through the stereotype of the ‘burden’ or ‘parasite’; establishing a normative comparison between ‘winners’, that is, those who have made an effort (studying, working, etc.) to gain access to a certain status or quality of life, and ‘losers’, that is, the free‐riders and welfare abusers who are unable to conform to neoliberal values of self‐sufficiency (Staerklé, 2009; Staerklé et al., 2012).

If you don't contribute anything, you are a burden. If you live in a society without doing anything, and you just consume and consume from it, you are a burden. If you were not here, society would make more progress. (P14)

On the other hand, those in favour of unconditionality confronted the stereotype of the ‘parasite’ by challenging narratives that limit social contribution to the narrow confines of the labour market, leaving out many essential activities (e.g. care work) (Pérez‐Orozco, 2019): ‘Not having a job means I don't contribute to society? That's very sexist; as if women who have spent their whole lives working for free in their homes haven't done anything to contribute…’ (P24). Moreover, not only was the ‘parasite’ stereotype questioned, but also the deservingness assessment itself (i.e. ‘Everyone, even a parasite, deserves to have decent living conditions’). In this way, equality is prioritized as a justice criterion superior to one's social contribution (i.e. equity), by appealing to the right to existence (Casassas, 2018; Raventós, 2021) of all human beings (i.e. inclusive group categorisation). Group membership is thus sufficient for access to common resources, and the distribution of rights and duties follows the communist maxim ‘from each according to their ability, to each according to their needs’ (communal sharing; Fiske, 1992).

I think it's fair, because a person, even if they're a slacker… Yeah, OK, but are you going to let them die? They have to sleep somewhere, they have to eat… (P20)

#### Sub‐theme 3.3. Autochthony‐based distribution: UBI Would have a pull effect

In contrast to the previous criteria, the autochthony criterion (Martinovic & Verkuyten, 2013; Verkuyten & Martinovic, 2017) appeals to group membership as a condition for receiving social assistance and guaranteed rights. Based on a social diversity conception of SO, a categorical differentiation is established between ‘nationals’ and ‘migrants’ (us vs. them) that is then objectified into the stereotype of the ‘racialized other’ (Staerklé, 2009; Staerklé et al., 2012), and used to legitimize autochthony beliefs, that is, that the first inhabitants of a territory should have more rights than newcomers (Martinovic & Verkuyten, 2013; Verkuyten & Martinovic, 2017). The objectification of migrants as ‘consumers’ of social assistance activates the threat of the pull effect of UBI and its consequences on the system's unsustainability, in line with realist threat theories (Stephan & Stephan, 2013). This fear thus translates into the imposition of residence and nationality criteria to access UBI, and other arguments regarding the need to implement a global UBI to avoid migratory movements.

If you just do it here, we'd suddenly become the most popular country, wouldn't we? I don't know how that would be controlled… I mean, how many of us can live here? How many millions can we fit? (P6)

In contrast, other participants oppose the autochthony criterion precisely because ‘that would exclude a lot of people who need it (i.e., migrants), who have no way of working and can't receive this type of aid’ (P2). (Out‐)group membership is recognized as a cause of unequal treatment and discrimination, providing the foundation for claiming group rights on the basis of group differences (Staerklé, 2009; Staerklé et al., 2012). Moreover, some participants argue that UBI could constitute a form of reparation to migrants for the historical (and ongoing) Western plunder of the global South (Hickel et al., 2021; Rodney, 1974). In this way, through perceived collective grievance and group injustice (van Stekelenburg & Klandermans, 2013), the stereotype of the ‘racialised other’ as a consumer of others' resources is countered by creating an image of ownership of those same resources, which serves to defend migrants' entitlement to UBI.

The money of this state is really our money, which is stolen from our countries. There are always a lot of prejudices, people who say that the migrant population comes here to collect benefits… Well, we are not even recovering what they have stolen from us, and they are still stealing. (P2)

### Theme 4: Feasibility in the current system

The fourth theme is built around the (un)feasibility of implementing a UBI, thereby referring to the broader social context in which the debate on this proposal is taking place. The current context is marked by a widespread social sense of powerlessness and hopelessness—defined by Fisher (2022) as capitalist realism—which limits political imagination and spreads the idea that there are no viable alternatives to capitalism (i.e. ‘It is easier to imagine the end of the world than the end of capitalism’). This results in a discursive tension between dystopian versus utopian re‐presentations of the future, which conditions the shared perception of the (un)feasibility of UBI, hindering versus promoting this joint project in the long term (Buhagiar & Sammut, 2023).

#### Sub‐theme 4.1. Unfeasibility: UBI Is impossible to implement

The rhetoric about the unfeasibility of UBI is objectified by generating images of chaos and collapse regarding its economic, political and cultural impact. For example, one of the core threats is that people would stop working if they had a guaranteed income, leading to a total collapse of the labour market and social organization as we know it: ‘It would be a bit chaotic, like Argentina, like Venezuela… On a global level, it would be a total economic collapse. We will go to barter in four days’. (P13). This creates the image of a dystopian future that arouses feelings of fear and urges the preservation of the current system, disregarding alternatives (Fisher, 2022). In this way, the stereotype of the ‘bad citizen’ (Hagerty & Barasz, 2020; Staerklé, 2009) serves as a warning of the dangers of UBI (i.e. people would waste it on drugs, whims or other non‐necessities), following a Moral order conception of SO (Staerklé, 2009; Staerklé et al., 2012). This enforces conformity with established social norms and legitimizes a system based on social control and disciplinary institutional intervention (Schroeder et al., 2017).

There are a lot of people who don't know how to manage their money… If you give everyone 900 euros a month, one goes on holiday and the other buys a TV. Especially lower class people… because they are not financially educated. Or people who have problems. Imagine with gambling, drugs, alcoholism… (P15)

Beyond these dystopian representations of the future, the unfeasibility of UBI is also constructed in terms of realism. Many participants believe that, even if UBI could be good philosophically, it is impossible to implement it in practice: ‘In a utopia, I would love for everyone to have a basic income, and not of 900, but of 1,500 euros, right? But, realistically, given the situation we are living in, it's complicated’ (P16). Thus, a distinction is drawn between the realistic and the utopian, resulting in the legitimisation of the status quo as inevitable (Jost, 2019; Jost & Banaji, 1994) and the perception of structural issues as intractable (Bird et al., 2024). This produces feelings of collective hopelessness and despair towards the future and installs an image of impossibility that paralyses action (Aubin et al., 2016; Bird et al., 2024); constituting an evident obstacle to moving this joint project forward.

I think housing is much more viable and they don't do it. They have been in government for four years: they talk a lot, but they do nothing… So, basic income is a chimera, it is absolutely impossible. (P23)

#### Sub‐theme 4.2. Towards utopia: UBI as a challenge to capitalism

In contrast to the impossibility discourse, an alternative narrative is constructed that not only re‐presents UBI as a necessary proposal, but also as an achievable one. To do so, UBI is re‐presented as a challenge to capitalism that could (1) contest capitalist values and practices in the present and (2) precipitate deeper social changes in the future (e.g. by decentralizing the importance of paid work) (Weeks, 2011). In this way, the challenge metaphor is able to make concrete this idea of UBI as a catalyst for social change, inspiring social hope and motivating collective action (van Stekelenburg & Klandermans, 2013).

It is a challenge to capitalism, because it is a departure from what capitalism says should be done: markets and multinationals controlling everything; the more work, the better; the more you buy, the better… I'm not saying it's an anti‐capitalist measure, but it's a challenge to it. (P24)

In this vein, participants objectify UBI as a tool for engaging in various forms of political activism, as well as for opening up new spaces in which to think about alternative ways of life. Here, utopian thinking—that is, the ability to positively imagine what the future society might look like—serves to challenge the view of the status quo as fixed and to foster the idea that social transformation is possible, inspiring greater confidence in the collective agency of organized society to effect change (Badaan et al., 2020; Fernando et al., 2018).

We would have the opportunity to think about other things, which we don't think about now, because we are stuck in the same old wheel… Maybe, as a society, we learn new values, right? Because, all of a sudden, there is a space for new values and ways of living. (P7)

## DISCUSSION AND CONCLUSIONS

Drawing on Staerklé (2009)'s model of SO and action‐oriented approaches to SRT (Buhagiar & Sammut, 2020, 2023), our research provides insight into how UBI is collectively re‐presented, incorporating a pragmatic emphasis on the ideological functionality of discourse. The reflexive thematic analysis we conducted captured (1) the shared values and normative beliefs in which the UBI debate is socially anchored; (2) the objectification of re‐presentations for UBI into different conceptions of distributive justice and group stereotypes; and (3) the social context in which this re‐presentation takes place. In this sense, our study advances previous literature by applying a socio‐constructivist theoretical framework (Sammut et al., 2015) to explain the collective elaboration of public opinions, meanings and beliefs about UBI.

Globally, our research showed that participants engaged in a rich discussion about UBI, although it was an unfamiliar idea to many of them; and they did so through anchoring and objectifying processes that helped to make the UBI proposal accessible and elaborate a political position on it (Staerklé, 2009; Staerklé et al., 2012). Specifically, the formation of social attitudes towards UBI was guided by different conceptions of SO (Staerklé, 2009; Staerklé et al., 2012). Importantly, these conceptions of SO were not individual nor isolated beliefs, but rather a network of socially established ideas that intermingled with each other, giving rise to complex—and sometimes ambiguous—political lay thinking (Staerklé, 2009). Moreover, following Buhagiar and Sammut (2020), this social re‐presentation for UBI was marked by competing ideological trajectories of hegemonic (system‐justifying) and alternative (system questioning) arguments and discourses, which crystallized into polemical social re‐presentations reflecting power dynamics and group conflicts. Concretely, our analysis suggested that the ideological anchoring of UBI revolves around the socially disputed values of equality and freedom.

Specifically, opposition to UBI is anchored through hegemonic system‐legitimizing beliefs (e.g. meritocracy, work ethic) (Jost, 2019; Jost & Banaji, 1994) that individualize aspirations for equality and freedom, making individuals responsible for their own destiny and thus rejecting the universal public provision of an income. This is in line with the results of previous survey studies on individual support for UBI (e.g. Baranowski & Jabkowski, 2021; Laenen & Gugushvili, 2023; Lim & Tanaka, 2019; Roosma & van Oorschot, 2020); as well as qualitative studies analysing public arguments around this proposal (Acuña Gómez et al., 2023; Herke & Vicsek, 2022; Rossetti et al., 2020; Zimmermann et al., 2020). In contrast, the joint advocacy for UBI is anchored in values of (structural) equality and (collective) freedom to contest this system‐legitimizing rhetoric and expose the injustice of the status quo, creating an alternative re‐presentation for UBI as a social right that should be guaranteed for all (Casassas, 2018; Raventós, 2021). In this sense, the social re‐presentation for UBI is constructed both in material and post‐material terms: as an aspiration towards (1) the equality of resources between groups and (2) the social emancipation and a livable life for the subordinate classes. Despite being widely present in the expert discourse on this proposal (see Afscharian et al., 2022), this dual ‘bread‐and‐roses’ re‐presentation has hardly been addressed in previous survey studies (see Schwander & Vlandas, 2020 for a notable exception). As for qualitative studies, arguments anchored in the shared value of equality (e.g. guaranteeing a basic floor, reducing inequality) dominate the social discourse (Acuña Gómez et al., 2023; Rossetti et al., 2020; Zimmermann et al., 2020); although some re‐presentations rooted in collective freedom (e.g. free time, emancipation from work) are also identified (Acuña Gómez et al., 2023; Zimmermann et al., 2020). Future studies could therefore address more comprehensively how different groups pragmatically manage this dual anchoring in different contexts, in order to advance the UBI project more effectively.

Besides, our analysis suggested that this tension between social values is objectified into group stereotypes (Staerklé, 2009; Staerklé et al., 2012) using deservingness judgements (van Oorschot, 2000) and distributive justice considerations (Veghte, 2016). Specifically, stereotypical images of undeserving groups in terms of need, reciprocity, and autochthony were created in order to oppose the UBI project; but these were also contested by appealing to an egalitarian principle of distributive justice. This equality principle is objectified using the human rights framework, which implies (1) a rupture with social categorisation and the adoption of an inclusive social identity that universalises the recipient of rights (Wenzel, 2004); (2) framing public resources as collectively owned and unjustly distributed, thereby justifying their egalitarian redistribution (van Stekelenburg & Klandermans, 2013); and (3) a social citizenship vision in which rights are granted to everyone for the mere fact of being alive, which defuses deservingness considerations (Dean, 2004). Previous literature has extensively addressed how deservingness beliefs shape policy attitudes in general (e.g. van Oorschot, 2000; van Oorschot et al., 2017), and even attitudes towards UBI in particular (e.g. Roosma & van Oorschot, 2020; Rossetti et al., 2020); however, studies focusing on how these deservingness considerations are challenged and a universalist lens is adopted are rather scarce. Thus, future research could delve deeper into people's reasoning around universality and the equal distribution of resources, a key issue regarding the social re‐presentation for UBI.

Finally, our analysis revealed that this meaning‐making process takes place in a social context marked by capitalist realism (Fisher, 2022) and fatalism about the future, which constitutes a major obstacle to moving the UBI project forward. This rhetoric regarding the unfeasibility of UBI has been identified in previous qualitative studies (Acuña Gómez et al., 2023; Herke & Vicsek, 2022; Rossetti et al., 2020; Zimmermann et al., 2020), but it has been largely neglected in quantitative survey studies. Therefore, this calls for a deeper analysis of the ways in which dystopian and fatalistic re‐presentations serve to limit our political imagination and insert the perception that implementing a UBI is impossible. Furthermore, following emerging literature on utopian thinking as a driver of collective action (e.g. Badaan et al., 2020; Fernando et al., 2018), our research suggests that re‐presenting UBI as a longer‐term strategy for challenging capitalism may be a tactic capable of countering fatalism and spreading feelings of hope and group efficacy (Weeks, 2011). As future‐oriented collective action is gaining prominence in political psychology research (e.g. Bosone et al., 2024; Milfont et al., 2014), how different re‐presentations for the future are collectively used to advance (vs. retreat) the UBI project could be a fruitful line for further research.

In sum, this study expands the theoretical understanding about how people collectively reason about UBI and how they construct shared meanings to argue for versus against this proposal. Despite focusing on a specific context (i.e. the Basque Country), we identified underlying socio‐psychological processes that can be applied to debates occurring in other territories. This reflects the cross‐cutting nature of the UBI discussion, which has to do with broader social issues such as (un)conditionality in access to rights or the centrality of (paid) work, among others. However, the generalisability and comparability of results are obviously limited, as this is qualitative research. Moreover, our research may have some limitations in terms of the study sample: (1) we may have incurred a self‐selection bias so that only participants interested in the UBI debate agreed to participate; and (2) probably our sampling did not sufficiently reflect some minoritised voices that could generate different UBI re‐presentations (e.g. people with a lower education level, migrants from the global South, disabled people, etc.). Nevertheless, we believe that our research makes both a theoretical and applied contribution, (1) by providing a theoretical framework on which to analyse the collective re‐presentation for UBI and (2) by offering argumentative strategies for pro‐UBI social movements to politically advocate for this proposal.

## AUTHOR CONTRIBUTIONS

**Itziar Guerendiain‐Gabás:** Conceptualization; investigation; writing – original draft; methodology; data curation; software; formal analysis. **Maitane Arnoso‐Martínez:** Conceptualization; investigation; writing – review and editing; supervision; methodology. **Lorena Gil de Montes:** Conceptualization; investigation; writing – review and editing; supervision; methodology.

## FUNDING INFORMATION

This research was funded by a pre‐doctoral grant awarded to Itziar Guerendiain‐Gabás by the University of the Basque Country (UPV/EHU) (PIF20/282). The authors would like to express their sincere thanks to all the participants who kindly agreed to be interviewed for this research. This study would not have been possible without their selfless contribution.

## CONFLICT OF INTEREST STATEMENT

The authors declared no potential conflicts of interest with respect to the research, authorship, and/or publication of this article.

## ETHICS STATEMENT

This research was conducted with the approval of the Ethics Committee for Research on Human Subjects (CEISH) of the University of the Basque Country (UPV/EHU) (M10/2020/278). We confirm that the research reported in this manuscript has been conducted in accordance with the APA Code of Conduct and the University of the Basque Country Research Ethics Committee. All participants consented to their participation in the study, in accordance with the provisions of the General Data Protection Regulation (EU‐2016/679). Results are reported honestly. The submitted work is original and not (self‐) plagiarized, and authorship reflects individual contributions.

## Supporting information


Tables S1“S3

## Data Availability

The data supporting the conclusions of this article (e.g. full interview transcripts, coded files, etc.) are available upon request from the corresponding author. The data are not publicly available to protect the privacy of the study participants.

## Footnotes

^1^
Conditional minimum income schemes are economic benefits provided by public administrations to guarantee a minimum income to households in situations of poverty and social exclusion. These programmes are typically characterized by (1) means‐testing (insufficient resources must be demonstrated to qualify for assistance); (2) household‐based regulation (the benefit amount varies according to the number of people in the household); and (3) job‐seeking conditions (the benefit is conditional on attendance at employment programmes, while refusal of job offers may be penalized by benefit withdrawal).

^2^
We provided participants with a fairly exhaustive definition of UBI, in order to evoke different debates surrounding this proposal (e.g. what constitutes a sufficient income, how such a proposal could be financed, etc.), and analyse how participants re‐elaborate this information and generate their own re‐presentations of UBI.

^3^
During the revision process, some themes and sub‐themes were further re‐elaborated. This resulted in slight changes in the proposed sub‐themes within Themes 2 and 4, in order to avoid redundancies and focus more clearly on the socio‐psychological processes involved (as opposed to the description of contents and meanings).

^4^
Amancio Ortega is a Spanish businessman, founder of the Inditex business group. In 2023, he became the first Spaniard to exceed 100 billion dollars in wealth, which places him in twelfth place in the ranking of the richest people in the world.

^5^
Ondarreta and Altza are two neighbourhoods in the city of Donostia (Gipuzkoa, Basque Country). According to the Basque Institute of Statistics (Eustat), Ondarreta is one of the richest neighbourhoods in the city, with an average household income of €68,770 per year. Altza, on the other hand, is a working‐class neighbourhood and one of the areas with the lowest income, with an average family income of €36,393.
