# Supplementary material for: Bread and Roses: Social re‐presentations for Unconditional Basic Income in the Basque Country
Source: Br J Soc Psychol. 2025 Jun 9;64(3):e12909. doi: 10.1111/bjso.12909 (PMC12149677; doi:10.1111/bjso.12909)
Supplement: Supplementary file 1 — Tables S1“S3 [file BJSO-64-0-s001.pdf]

Online Appendix of “Bread and Roses: Social Re-presentations for Unconditional Basic Income in the Basque Country”

Table S1. Complete Sociodemographic Characteristics of Each Study Participant

Table S2. Full Interview Script

Table S3. Results from Reflexive Thematic Analysis Including Original Quotations in Spanish

Table S1

Complete Sociodemographic Characteristics of Each Study Participant

	Gender	Age	Migrant background	Educational level	Employment status	Job position	Type of contract	Type of workday	Nº of persons in the household	Dependents	Monthly household income	Subjective social class	Political stance	Vote in the last elections	Union membership	Participation in social movements	Information level on UBI	Stance on UBI
P1	Man	35-44 years	No	Secondary	Employed	Delivery man	Permanent	Full-time	1	No	1001-1500€	Lower	Extreme left	SUMAR	No	No	High	In favour
P2	Woman	25-34 years	Sahara	Vocational training	Employed	Community worker	Permanent	Full-time	2	No	2001-2500€	Lower	Extreme left	No voting rights (EH Bildu)	ELA	Anti-racism, feminism, social integration	Medium	In favour
P3	Woman	45-54 years	No	University degree	Employed	Shop assistant	Temporary	Part-time	3	Yes	2501-3500€	Lower-middle	Left	SUMAR	No	No	Low	Against
P4	Man	35-44 years	Germany	University degree	Employed	Teacher	Temporary	Part-time	1	No	1001-1500€	Middle	Centre	Did not vote	No	No	Low	In favour
P5	Man	55-64 years	No	Master's degree	Employed	Technical manager	Permanent	Full-time	5	No	5001-7000€	Lower-middle	Left	SUMAR	No	No	Low	In favour
P6	Man	45-54 years	No	PhD	Employed	Teacher	Permanent	Full-time	2	No	3501-5000€	Middle-upper	Centre-left	EH Bildu	LAB	Ecologism, independentism	Low	In favour
P7	Non-binary	25-34 years	No	Master's degree	Employed	PhD researcher	Temporary	Full-time	5	No	3501-5000€	Middle	Left	EH Bildu	No	Queer, feminism	Low	In favour
P8	Woman	25-34 years	No	University degree	Employed	Software developer	Permanent	Full-time	1	No	1001-1500€	Middle-upper	Centre-right	PP	No	No	Low	Against
P9	Woman	35-44 years	No	University degree	Household and care work				4	Yes	2501-3500€	Middle-upper	Left	SUMAR	No	No	Medium	In favour
P10	Man	35-44 years	No	Master's degree	Employed	Project manager, Entrepreneur	Permanent, Self-employed	Full-time	3	Yes	More than 7001€	Middle-upper	Centre-right	Did not vote (EAJ-PNV)	No	No	Low	Against
P11	Man	18-24 years	No	University degree	Student				3	No	2001-2500€	Lower-middle	Extreme left	Did not vote	No	Yes (unspecified)	Medium	Against
P12	Man	25-34 years	No	University degree	Employed	Administrative assistant	Temporary	Full-time	3	No	More than 7001€	Middle-upper	Centre	Did not vote	No	No	Medium	Against
P13	Woman	55-64 years	No	University degree	Employed	Accountant	Permanent	Full-time	3	No	5001-7000€	Middle	Centre	UPN	No	No	Medium	Against
P14	Man	18-24 years	No	University degree	Student				3	No	1501-2000€	Middle	Centre-right	EAJ-PNV	No	No	Medium	Against

P15	Man	25-34 years	No	Master's degree	Employed	Assistant director	Permanent	Full-time	2	No	2501-3500€	Middle-upper	Centre-right	EAJ-PNV	No	No	Low	NA
P16	Woman	45-54 years	No	Master's degree	Employed	Teacher	Temporary	Full-time	3	Yes	3501-5000€	Middle	Left	NA	Yes (unspecified)	No	Low	NA
P17	Woman	35-44 years	No	PhD	Employed	Nutritionist	Permanent	Full-time	1	No	1501-2000€	Middle	Centre-right	Did not vote	No	No	Low	In favour
P18	Non-binary	25-34 years	No	Master's degree	Employed	Community worker	Permanent	Part-time	8	No	2501-3500€	Middle	Extreme left	Did not vote (EH Bildu)	ESK	Autonomism	Low	In favour
P19	Woman	25-34 years	Romania	Master's degree	Unemployed				2	No	701-1000€	Lower	Left	No voting rights (SUMAR)	ESK	Feminism	High	In favour
P20	Man	55-64 years	No	University degree	Employed	Maintenance assistant	Permanent	Full-time	1	No	1501-2000€	Middle	Left	EH Bildu	LAB	No	Medium	In favour
P21	Man	55-64 years	No	University degree	Retired	Teacher			4	No	3501-5000€	Middle	Left	SUMAR	No	No	Low	NA
P22	Woman	55-64 years	No	Vocational training	Employed	Cleaner	Permanent	Part-time	3	No	2001-2500€	Lower-middle	Left	PSOE	UGT	No	Low	In favour
P23	Man	55-64 years	No	Secondary	Retired	Firefighter			3	No	2501-3500€	Lower	Extreme left	SUMAR	CNT	No	Medium	In favour
P24	Woman	55-64 years	No	University degree	Employed	Administrative assistant	Permanent	Full-time	2	No	3501-5000€	Middle-upper	Extreme left	EH Bildu	ESK	Internationalism, feminism, migrations	High	In favour
P25	Man	55-64 years	No	University degree	Employed	Medical surgeon	Permanent	Full-time	4	No	NA	Middle-upper	Right	PP	No	No	Medium	Against
P26	Man	25-34 years	Russia	Vocational training	Employed	Ambulance driver	Temporary	Full-time	2	No	2501-3500€	Middle	Centre	No voting rights	No	No	Low	In favour

Note. NA = No answer. When the person did not vote in the last election (for whatever reason), the party preference (when provided) is shown in brackets.

Table S2

Full Interview Script

**Preliminary questions**
Sociodemographic data
– Gender<br>– Age<br>– Migrant background<br>– Educational level<br>– Employment status (*Employed persons: job position, type of contract, type of workday)<br>– Number of persons in the household<br>– Dependents (yes/no)<br>– Monthly household income<br>– Subjective social class<br>– Political stance<br>– Party voted for in the last elections<br>– Union membership<br>– Participation in social movements
**Main questions**
Definition of UBI
"Lately, there has been more and more talk about the **Unconditional Basic Income** proposal. This proposal consists of the following: The government would pay a **monthly income to all people** living in our country. The aim is to guarantee everyone an **adequate standard of living**, so the amount of the basic income should be at least above the poverty line. This income would be given **individually** to each person, not to families or cohabitation

units. It would be given to everyone, **without any kind of requirement or condition**. In other words, it would be paid regardless of whether or not the person is rich or poor, whether or not they are employed, etc. This measure would be **financed through taxes**, so that the richest people (although they would also receive the basic income) would be the ones who would have to contribute the most in taxes to finance it."

Arguments/positioning on UBI

- What comes to your mind when you hear about UBI? What is the first thing you thought of when I read the definition?
- What are your views on this proposal? Would you be for or against the implementation of a UBI?
- What would be your arguments in this regard? What arguments in favour of UBI can you think of? And what arguments against it?
- What advantages and/or disadvantages do you think this proposal could have? What do you think would happen if a UBI were to be implemented in our society? What consequences do you think this could have on a social level?
- What doubts do you have about this? What aspects are you not convinced about …? What kind of problems do you think might arise?

Complementary questions (why questions)

- Why do you think/believe that? On what basis do you think/believe that? On what basis do you make that argument?

Summary questions

- In summary, you have told me that you think X and Y, is that right? So, in summary, would you say that your arguments for/against UBI are X and Y?
- Do you think this summary captures your views well, or is there anything you would like to clarify? Is there anything else we haven't talked about that you think is important to point out/emphasise? Is there anything else you would like to add?

Final questions

- Previous level of information on UBI
- Explicit stance for/against UBI

Table S3

Results from Reflexive Thematic Analysis Including Original Quotations in Spanish

Theme/ Subtheme/ Code	Quote	
	English	Spanish
**1. Origins of Social Inequality**		
1.1 Inequality as an Individual Issue: Those Who Earn a Lot, It's Because They Work a Lot		
Poverty as an individual issue	"Take away the subsidies. If you are fit to work, you work. There is no aid. That's the only way. Either that, or the differential should be much higher; basic income should not be above the poverty line, but it should be a minimum allowance, so that you can eat a bowl of soup every day and that's it." (P13)	"Quitar las ayudas. Si eres apto para trabajar, trabajas. No hay ayuda. Es la única forma. Eso, o que el diferencial sea mucho mayor; que la renta básica no sea por encima del umbral de pobreza, sino que sea una ayuda mínima, para que puedas comer un plato de sopa cada día y punto-pelota." (P13)
	"We have to support the people who have the least, but carefully, because in the end excessive subsidies create dependent people. What we have to try to do is to give people quality jobs, not subsidies." (P25)	"Al final las subvenciones excesivas… Hay que apoyar a la gente que menos tiene, pero con cuidado, porque al final se crea gente dependiente. Lo que hay que procurar es que la gente tenga trabajo de calidad, no subvenciones." (P25)
Wealth as an individual issue	"I don't think anyone is given free money: those who earn a lot, it's because they work a lot and it takes up a lot of time. Maybe there is a 1%, the football player, the lucky one, Amancio Ortega... but I don't think it is representative. Of the remaining 99% of mortals, those who earn a lot, it is because it takes a lot of time and effort." (P10)	"No creo que a nadie le regalen el dinero: el que gana mucho, es porque se esfuerza mucho y le consume mucho tiempo. A lo mejor hay un 1%, el futbolista, el que tenga mucha suerte, el Amancio Ortega de turno… pero no me parece algo representativo. Del 99% restante de los mortales, el que gana mucho, es porque le consume mucho tiempo y esfuerzo." (P10)
1.2. Inequality as a Structural Issue: There are Poor People Because There are Very Rich People		

Poverty as a structural issue	"I think poverty is a crime: to be born in Ondarreta or in Altza, and for the difference in mortality to be eight years, I think it's a fucking crime. In Ondarreta, people have excellent bodies, because they have resources, jobs as lawyers, university professors, executives... And then I go to Altza and I see another type of body... And I think it's a fucking crime." (P6)	"La pobreza me parece un crimen: nacer en Ondarreta o en Altza, y que la diferencia de mortalidad sea ocho años, me parece un puto crimen. En Ondarreta, están los cuerpos excelentes de quienes tienen recursos, con trabajos de abogados, profesores de universidad, empresarios… Y luego voy a Altza y veo otro tipo de cuerpos… Y eso me parece que es un puto crimen." (P6)
Wealth as a structural issue	"It's indecent how few people have so much money. Because the people who really have money and power... don't have it because they've worked hard for it. If Botín or Amancio Ortega's daughter have a lot of money, it's not because they've worked for it." (P1)	"Es indecente lo de que poca gente tenga tanta pasta. Porque la gente que tiene realmente dinero y poder y todo eso… no lo tiene porque se lo haya currao. Si Botín o la hija de Amancio Ortega tienen un montón de pasta, no es porque se lo han currao." (P1)
Universal right to a dignified life	"Everyone has the right to live with dignity. And to live with dignity in this world, we need money. Because that's how it works. So, I think it's fair. With a minimum of empathy, you should see that we all deserve that just because we live in this shitty society." (P7)	"Todo el mundo tiene derecho a vivir dignamente. Y para vivir dignamente en este mundo, necesitamos dinero. Porque funciona así. Entonces, ya está, me parece justo. Con tener un mínimo de empatía, deberías ser capaz de ver que todes nos merecemos eso solo por estar vives en esta sociedad de mierda." (P7)
UBI as an extension of social rights	"We are all in favour of public health care, which is a distribution of wealth. We are in favour of public education. And this is another distribution of wealth. A basic income would be an improvement in other aspects, the right to live in greater dignity." (P24).	"A todo el mundo nos parece bien que exista una sanidad pública, que eso es un reparto de la riqueza. Nos parece bien que exista una educación pública. Y entonces esto es otro reparto de la riqueza. Una renta básica sería una mejora en otros aspectos, el derecho a vivir de una manera un poco más digna" (P24)

2. The Right to a Good Life

2.1. Freedom as a Collective Issue: We Would Feel Freer To Choose

Freedom as a collective issue	"In this village, since we were 14 years old, we have all had to work. All of us. Employers came to your house to tell you: 'you have a job in the factory'. And there were no other options. So,	"En este pueblo, desde que teníamos 14 años, hemos tenido que trabajar. Todos. Los empresarios venían a tu casa a decir, 'esta niña tiene puesto en la fábrica'. Y no ha habido más opciones.

	with basic income all that would end, wouldn't it? We would feel freer to choose." (P22)	Entonces, claro, con esta renta, todo eso se acabaría, ¿no? Nos sentiríamos más libres de elegir.” (P22)
Emancipation from work	"We should all be guaranteed the minimum: food, housing... and then, if you want to work and buy a car, you work and buy a car with a decent salary, but nobody is going to tell you that you have to work to eat." (P1)	“Deberíamos tener todos asegurado un mínimo consumo, una vivienda, comida… y luego, si quieres trabajar y comprarte un coche, pues trabajas y te compras un coche con un sueldo digno, pero nadie te va a decir que tienes que trabajar para comer.” (P1)
Emancipation of women	"UBI would give me more decision-making power, I would feel less tied to having to get this money from somewhere. Now it's a dependency on my husband, otherwise it would be a dependency on my company... I envision more independence to be able to choose, right?" (P9)	“La RBI me daría más poder de decisión, me sentiría menos atada a tener que conseguir este dinero de algún medio, ¿no? Ahora es una dependencia con mi marido, si no, sería una dependencia con mi empresa … Visualizo más independencia para poder elegir, ¿no?” (P9)
Slowing down the pace	"Maybe people would work part-time to spend more time with their family, with their friends, to have a more leisurely life... because they know they have that extra 1,000 euros." (P3)	“Igual la gente, trabajaría media jornada para estar más con su familia, con sus amigos, tener una vida más ociosa… porque sabe que tiene esos 1.000 euros de extra.” (P3)
	"You could afford to spend an hour or two with your friend calmly and then go shopping without rushing. Live more slowly." (P19)	“Podrías permitirte ir tranquilamente a estar una hora o dos horas con tu amiga, y luego ir a hacer la compra sin prisas. En ese sentido, bajar el ritmo.” (P19)
Time for activities beyond paid work	"And also do some work to help the elderly, the children... Helping the neighbourhood. I'd like to create a community among the neighbours, to help this one who is disabled, the other one who is older..." (P22)	“Y hacer algo de trabajo también de ayuda a ancianos, a niños… Ayudar al vecindario. Ojalá se crearía, entre los propios vecinos una comunidad, una ayuda para el del primero que se ha caído y está inválido, al otro que está anciano...” (P22)
	"There would also be more time for social mobilisation. There are many people who, because of their work rhythms, only participate in specific moments of mobilisation, such as a demonstration. If we didn't have those rhythms, perhaps we could get more involved in our day-to-day lives." (P18)	“Para la movilización social también habría más tiempo. Hay una masa grande de gente que, por los ritmos de trabajo que lleva, solo participa en momentos puntuales de movilización, como puede ser una mani. Si no tuviéramos esos ritmos, igual podríamos estar más implicadas en cada causa en nuestro día a día.” (P18)

Improved mental health	"It would take away the pressure that a lot of people have of not making ends meet, because you have a shit salary, and it doesn't allow you to have a normal life. And you can't think about anything else but that.... If you take that part out, people would live a much better life." (P1)	“Quitaría la presión que tiene mucha gente, de no llegar, porque tienes un sueldo de mierda, y no te llega para poder tener una vida más o menos normal. Y no puedes pensar en otra cosa que no sea llegar a fin de mes o endeudarte… Si quitas esa parte, la gente viviría mucho mejor.” (P1)
Development of post-materialistic needs	"Because if you have your basic needs covered, you can open your mind to new horizons, right? If you're always looking for money to pay the mortgage, you're not going to start thinking about doing a project for a bike path that produces energy, you know? In that sense, I think it can be a driving force for our society." (P19)	“Porque si tienes las necesidades básicas cubiertas, puedes abrir tu mente a nuevos horizontes, ¿no? Si estás todo el rato pendiente de conseguir dinero para pagar tu hipoteca, pues no te vas a poner a pensar en hacer un proyecto de un carril bici que produzca energía, ¿sabes? En ese sentido, creo que puede ser un motor para nuestra sociedad.” (P19)
Improving social and community relations	"Maybe there was even less crime, less robbery, because we already have the minimum, right? If we are covered, what is the need to rob people?" (P26)<br><br>"At the neighbourhood level, I think the street would be more inhabited. Right now, the streets are made to go to work, as a place to pass through. If we don't need that, I think the spaces would be inhabited to be, to talk, to live." (P18)	“Igual hasta existía menos delincuencia, menos robos, porque ya tenemos lo mínimo, ¿no? Si estamos cubiertos, ¿qué necesidad tienes de robar a la gente?” (P26)<br><br>“A nivel del barrio, creo que la calle estaría más habitada. Ahora mismo, las calles están hechas para ir al trabajo, o sea, como de paso. Si no necesitamos ese lugar de paso, creo que los espacios se habitarían para estar, para hablar, para vivirlos.” (P18)
2.2. Freedom as an Individual Issue: I Would Like to be Financially Independent		
Freedom as an individual issue	"I would like to be financially independent, and be able to live without working; but not living by taking advantage of others. Instead, I want to collect rents or invest money so that I can get there. But not on the basis that others have to make a much greater effort than me so that I can stay at home." (P16)<br><br>"I think that what I would do would be to try to find sources of funding, sources of income, personal, private, making investments... I am aware that not everyone can do it, but well... Those of us who can, in some way, I think we have to try to go	“A mí me gustaría ser económicamente independiente. Y poder no trabajar, pero no vivir de los demás, aprovechándome de los demás; sino teniendo rentas o invirtiendo dinero, para poder llegar a esa situación, pero no asumiendo que otras personas tienen que hacer un esfuerzo muchísimo más grande que yo, para que yo pueda estar en casa.” (P16)<br><br>“Yo considero que yo lo que haría sería intentar buscar fuentes de financiación, fuentes de ingresos, personales, privadas, haciendo inversiones… que soy consciente de que no todo el

	that way. And yes, there are people who can't, or who will never be able to, so they have to try elsewhere, right? But, well, personal effort has to be reflected at some point. I think it's good for society." (P16)	mundo puede hacerlo, pero bueno… Los que podemos, de alguna manera, creo que tenemos que intentar ir por ahí. Y sí que hay gente que no puede, o que nunca va a poder, pues tienen que intentarlo por otros lados, ¿no? Pero bueno, que el esfuerzo personal tiene que verse reflejado en algún momento. Creo que es bueno para la sociedad." (P16)

3. Accessibility: Who Should Get a UBI and Who Should Not

3.1. Need-Based Distribution: A Basic Income for Those Who Need it

UBI only for those who need it	"A couple with one child is not the same as a couple with four children.... But I'm already talking about situations of vulnerability, when one is ill, when the mother of another comes to look after him... Helping these people on a case-by-case basis. But an uncontrolled distribution, for everyone? No, I'm not in favour of that." (P3)	"Claro, no es lo mismo una pareja con un hijo, que con cuatro hijos… Pero me voy ya a situaciones de vulnerabilidad, que uno está enfermo, que a otro viene la madre a cuidarle desde… O sea, ayudar a esa gente viendo caso por caso. Pero así una renta sin control, ¿pa' todo el mundo? No, no estoy muy a favor." (P3)
Rich people should not get an UBI	"Yes, we could guarantee a basic income for all... But problems arise. Why are you going to give 800 euros to Amancio Ortega? He doesn't need it, does he? And neither do I..." (P25)	"Sí, podríamos garantizar una renta básica para todo el mundo… Pero, claro, te surgen problemas. ¿Por qué le vas a dar 800 euros a Amancio Ortega? Que no necesita, ¿no? Ni a mí, que tampoco los necesito…" (P25)
Limitations of conditional minimum income schemes	"I was talking to a friend of mine and she was telling me that, when it comes to applying for aid, you almost have to do a university degree. So how many people can't access these benefits? Because she is helping her work colleague, who is South American, and the poor thing doesn't know anything about it... There is a lot of aid that doesn't reach everyone, especially the people it should reach." (P3)	"El otro día estuve hablando con una amiga y decía que, a la hora de solicitar ayudas, dice que casi tienes que hacer una carrera universitaria. Entonces, ¿cuánta gente no puede acceder a estas ayudas? Porque ella está ayudando a una chica que trabaja en el bar, sudamericana, que la pobre no se entera… O sea, hay muchas cosas que no llegan a todo el mundo, a gente que debería llegar, además…" (P3)
Advantages of UBI's universality	"In that respect, it's very inclusive for everyone, isn't it? It would cover a thousand situations of people who right now don't have access to any aid, and in that case, yes, we would all have access." (P17)	"En ese aspecto, es muy inclusivo para todos, ¿no? Abarcaría mil situaciones de gente que ahora mismo no tiene acceso a ninguna ayuda, y en ese caso, sí que tendríamos todos acceso." (P17)

	"If we all received it, things would change. When only a few receive it, they are discriminated against. Because the rest have to get it with sacrifice. But if they give it to everyone, I think it would be easier." (P22)	"Claro, si ya lo recibiéramos todos, cambiaría. Cuando son unos pocos los que la reciben, se les discrimina. Porque el resto lo tiene que conseguir a base de sacrificio. Pero si se lo das a todo el mundo, yo creo que sería más fácil." (P22)

3.2. Reciprocity-Based Distribution: If You Want to Receive, You Have to Give Something in Return

If you want to receive, you have to give something in return	"Everyone has rights and obligations in this society. Some people seem to have only rights and no obligations. OK, you have the right to get this, but... What good are you going to do for society?" (P16)<br><br>"I really believe that if a person can work, they should work. Not only on a personal level, but also thinking about society, about others… You have to work." (P8)<br><br>"Clean the roads, clean the mountains, accompany the elderly, help, you know? I mean, ask for something in return. There are many things people can do." (P25)	"Todo el mundo tenemos derechos y obligaciones, como sociedad que somos. Hay gente que parece que solo tiene derechos, que no tiene obligaciones. Vale, tú tienes un derecho de cobrar esto, pero… ¿Qué bien vas a hacer a la sociedad?" (P16)<br><br>"Yo creo que realmente si una persona puede trabajar, debería hacerlo. No sólo a nivel personal, sino también pensando en la sociedad, en los demás... Deberías trabajar." (P8)<br><br>"Limpiar las carreteras, los montes, que acompañe a ancianos, que ayude, ¿sabes? Es decir, exige algo a cambio. Hay muchas cosas que se pueden hacer." (P25)
If you contribute nothing, you are a liability/ parasite	"It's all very well to take advantage of society, but if you don't contribute anything, you are a burden. If you live in a society without doing anything, and you just consume and consume from it, you are a burden. If you were not here, society would make more progress." (P14)<br><br>"There are times when you can't work and there are times when you can't get a job, which are different things, aren't they? If you can't work because you have a psychological or physical disability, that's fine." (P10)	"Es muy bonito aprovecharte de la sociedad, pero si no aportas nada, eres un lastre. Porque si tú estás viviendo en una sociedad sin aportar nada, y solo consumes y consumes de ella, eres un lastre. Si tú no estuvieras aquí, la sociedad avanzaría más." (P14)<br><br>"Hay veces que no puedes trabajar y hay veces que no puedes conseguir un trabajo, que son cosas diferentes, ¿no? Si tú no puedes trabajar, porque tienes una discapacidad psicológica o física, no pasa nada." (P10)
The right to a dignified life	"I think it's fair, because a person, even if they're a slacker... Yeah, OK, but what are you going to do? Are you going to let them die? They have to sleep somewhere, they have to eat..."	"Yo lo considero justo, porque una persona, aunque sea un vago… Ya, vale, pero, ¿qué vas a dejarlo, que se muera? Hay que dormir en algún sitio, hay que comer…" (P20)

is superior to contribution	(P20)	
Social contribution goes beyond market productivity	"Not having a job means I don't contribute to society? That's a lie, and it's also very sexist. It's like saying that women who have spent their whole lives working for free in their homes have contributed nothing to society. My mother has contributed nothing to society? Fuck you." (P24)	“¿No tener un empleo significaría que no voy a aportar a la sociedad? Eso es una falacia y, además, muy machista. Es como decir que las mujeres que han estado trabajando gratis toda la vida en sus casas, no han aportado nada a la sociedad. ¿Mi madre no aportó a la sociedad? Vete a tomar por culo.” (P24)

3.3. Autochthony-Based Distribution: UBI Would Have a Pull Effect

UBI would have a pull effect	"Obviously, if you just do it here, we'd suddenly become the most popular country, wouldn't we? I don't know how that would be controlled... I mean, how many of us can live here? How many millions can we fit?" (P6)	“Si solo lo haces aquí, pues, evidentemente, de repente pasaríamos a ser el país más popular, ¿no? No sé cómo se tendría que controlar eso, ¿no? O sea, ¿cuántos podemos llegar a vivir aquí? ¿Cuántos millones cabemos?” (P6)
UBI should be implemented globally	"Put it universally, not just here. Because then, 'OK, I'll move there', but it can't be, let everybody come... Then we generate another kind of problem. It has to be a very, very broad territory, and with the purpose of deploying it all over the world." (P5)	“Ponerlo de forma universal, no solamente aquí. Porque, entonces, ‘vale, yo me mudo allí’, pero claro, no puede ser, permitir que todo el mundo venga… Entonces generamos otro tipo de problema. Tiene que ser un territorio muy, muy amplio, y con el propósito de desplegarlo a todo el mundo.” (P5)
Migrants should have the right to UBI	"I don't think it's right to demand a residency requirement, that would exclude a lot of people who need it, who have no way of working and can't receive this type of aid. Basic income would be fine with me, as long as migrants are not required to provide documentation." (P2)	“No me parece bien exigir un requisito de residencia, eso excluiría a muchísima gente que lo necesita, que no tiene forma de trabajar, y tampoco puede recibir este tipo de ayudas. La renta básica me parecería bien, siempre y cuando no tenga requisito de exigir documentación a las personas migrantes.” (P2)
	"What really gets on my nerves is this 'pull effect'... It seems to me to be a fascist argument, as if we were suddenly afraid that the Basque Country would turn black." (P18)	“Lo que me chirría mucho es lo del ‘efecto llamada’… Me parece un argumento fascista, como si de repente tuviéramos miedo a que Euskal Herria se convirtiera en negra.” (P18)

UBI as a form of reparation to migrants	"The money of this state is really our money, which is stolen from our countries. There are always a lot of prejudices, people who say that the migrant population comes here to collect benefits... Well, we are not even recovering what they have stolen from us, and they are still stealing. It would take centuries for them to give me back what they are stealing from me." (P2)	"En realidad, el dinero que hay en este Estado es nuestro dinero, que nos lo roban de nuestros países. Siempre hay muchos prejuicios, gente que dice que la población migrante viene aquí a cobrar ayudas… Bueno, es que ni estamos recuperando lo que se nos ha robado, y que se nos sigue robando. O sea, tardarían siglos en devolverme lo que me están robando." (P2)

4. Feasibility in the Current System

4.1. Unfeasibility: UBI is Impossible to Implement

There is not enough money	"Let's see who pays that in Spain. Because it looks very cool, but 900 euros for 50 million... well, do the maths, see how much that would cost. Who would pay for it? Money doesn't come out on its own, that's the mentality that a lot of people have, that money comes out on its own..." (P15)	"A ver quién paga eso en España. Porque queda muy guay, pero 900 euros por 50 millones... pues hazte el cálculo, a ver qué gasto sería para las arcas del Estado. ¿Quién lo pagaría? El dinero no sale solo; esa es la mentalidad que tiene mucha gente, que el dinero sale solo…" (P15)
	"I always say that there is a spending pie. If everybody gets 900 euros, the pie runs out of slices. How would you pay for education, health care, social benefits...?" (P10)	"Siempre digo que hay un bizcocho de gasto. Si todo el mundo recibe 900 euros, el bizcocho se queda sin partidas. ¿Cómo pagarías la educación, la sanidad, ayudas sociales…?" (P10)
The system can't be sustained if people don't work	"If a lot of people don't work, the economy goes down the drain. If there is no production economy, you tell me, how are we going to survive, and how is the state going to pay for all that? It would be a bit chaotic, like Argentina, like Venezuela… On a global level, it would be a total economic collapse. We will go to barter in four days." (P13)	"Si hay mucha gente que no trabaja, la economía se va al traste. Si no hay economía de producción, ya me dirás tú, cómo se va a sobrevivir, y cómo va a pagar el Estado todo eso. Sería un poco caótico, como Argentina, como Venezuela… A nivel mundial, sería el colapso económico total. Pasaríamos al trueque en cuatro días." (P13)
Devaluation of the work ethic	"With that basic income, a lot of people may not strive for more. And if there is not an entrepreneurial mentality, a passionate mentality, of doing things for the good of all; that can lead to bad things." (P15)	"Si tienes esa renta básica, mucha gente puede no esforzarse por conseguir algo más. Y si no hay una mentalidad emprendedora, pasional, de hacer cosas por el bien de todos; eso puede llevar a mal." (P15)

Promoting inappropriate consumption	"There are a lot of people who don't know how to manage their money... You have to measure it very well, because if you give everyone 900 euros a month, one goes on holiday and the other buys a TV. Especially lower class people... because they are not financially educated. Or people who have problems. Imagine with gambling, drugs, alcoholism... I don't even want to imagine it, it would create a lot of problems." (P15)	"Hay mucha gente que no sabe administrarse el dinero... Entonces, hay que medirlo muy bien, porque si a todo el mundo ahora le das 900 euros al mes, uno se va de vacaciones y el otro se compra una tele. Sobre todo la gente de clase baja… porque no tienen educación financiera. O gente que tiene problemas. Imagínate con la ludopatía, las drogas, el alcoholismo… Es que no me quiero ni imaginar, crearía muchos problemas." (P15)
It is impossible to achieve wealth redistribution	"Then there is the bag of legal fraud, which are the people who can avoid paying because their economic level and their legal advice allows them to pay less, much less. So, those in the middle are going to pay, they are going to pay their own subsidy, that of the poor, and that of the rich." (P21)	"Otra cosa es la bolsa de fraude legal, que son las personas que pueden evitar pagar, porque su nivel económico y de asesoría jurídica permite que paguen menos, mucho menos. Entonces, van a pagar los de en medio, van a pagar su subsidio propio, el de los pobres, y el de los ricos." (P21)
	"If everyone received the basic income, things would be more expensive and it would be useless to get it... The rent would be 2,000 euros. So, a person who can't pay a rent of 1,000 euros now, wouldn't be able to pay a rent of 2,000 euros either, you know?" (P8)	"Si todo el mundo lo cobrase, igual las cosas son más caras y no sirve para nada a cobrarlo… El alquiler pasaría a ser 2.000 euros. Entonces, una persona que no puede pagar ahora un alquiler de 1.000, tampoco podría pagar uno de 2.000, ¿sabes?" (P8)
There are resources, but no political interest	"The left has a lot of pretence. Basic income, we are all equal, I don't know what... These are things that are all very well for a hearing in parliament, but on a day-to-day basis, who deals with these things? You don't see that in the grassroots, in the real people." (P3)	"La izquierda tiene mucho postureo. La renta básica, que todos somos iguales, que no sé qué… Hay cosas que dices, está muy bien para una audiencia en el Parlamento, pero en el día a día, ¿quién se encarga de esas cosas? Eso luego no se ve en la gente, en la base, en el pueblo real." (P3)
	"I think housing is much more viable and they don't do it. They have been in government for four years: they talk a lot, but they do nothing… So, if they are not able to provide housing for people who do not have it, basic income is a chimera, it is absolutely impossible." (P23)	"Me parece mucho más viable lo de la vivienda y no lo hacen. Ya llevan cuatro años gobernando, se llenan la boca, pero no... Entonces, si no son capaces de dar una vivienda a la gente que no tiene, pues la renta básica es una quimera, es absolutamente imposible." (P23)

The economic elites will not allow it	"I see it as very difficult to convince those who could apply it, on the one hand, and then, those who could apply it, even if they were convinced, would have a lot of resistance from the financial powers." (P20)<br><br>"And there are people in power who would work against it, because they would think 'careful, let people do what they want? no, because I'll stop earning what I'm earning, exploiting everybody'." (P5)	“Veo muy difícil convencer a los que pudieran aplicarlo, por un lado, y luego, los que pudieran aplicarlo, aunque estén convencidos, tendrían mucha resistencia por parte de los poderes financieros...” (P20)<br><br>“Y hay poderes que obrarían en contra, porque pensarían ‘cuidado, ¿que la gente haga lo que quiera?, no, porque si no, yo dejo de ganar lo que estoy ganando, explotando a todo el mundo’.” (P5)
UBI would actually reinforce capitalist system	"In practice, it's about being cheated. They're going to cheat you so that... well, so that the people they are going to expel from the labour process don't starve. And, at the same time, it serves as an ideological mechanism to sustain capitalism. Because it will generate confidence in the state, which is going to give you that little payment, at a time when the delegitimisation of the state is in the interests of the labour movement, in my opinion." (P11)	“Prácticamente, esto es una cuestión de que te la van a colar. Te la van a colar para poder... pues eso, que no se te muera de hambre la gente que van a expulsar del proceso de trabajo. Y aparte, que valga como mecanismo ideológico de sustentar el capitalismo. Acaba por generar una mínima confianza en el Estado, que es quien te va a dar esa paguita, en un momento en el cual la deslegitimación del Estado es una cosa que interesa al movimiento obrero, para mí.” (P11)
Social conservatism	"I think it is a very good idea, but society is not ready for it. Because the election results confirm it. We are going backwards instead of forwards. They think that subsidies are too much, imagine a basic income..." (P23)<br><br>"Then there is the ideological part, of liberal people who say 'no, everyone gets what they work for'. Like people who are not in favour of public health and education, right? Those people are there, you know, that's why I think it's difficult to do it." (P20)	“Me parece una idea buenísima, pero no está la sociedad preparada, no. Porque los resultados electorales así lo confirman. Vamos hacia atrás en vez de hacia Adelante. Les parecen demasiado las ayudas, como para una renta básica…” (P23)<br><br>“Luego está la parte ideológica, de gente liberal que digan ‘no, aquí cada uno lo que trabaje’. Como la gente que no está por la sanidad pública y la educación pública, ¿no? Esa gente está ahí, ¿sabes?, por eso lo veo difícil de hacer.” (P20)
4.4. Towards Utopia: UBI as a Challenge to Capitalism		
UBI questions capitalist values	"It is a challenge to capitalism, because it goes outside the capitalist channels. It is a departure from what capitalism says should be done: markets and multinationals controlling	“Es un desafío al capitalismo, porque se sale de los cauces capitalistas. Se sale de lo que el capitalismo dice que hay que hacer: que sean los mercados y multinacionales quienes

	everything; the more work, the better; the more you buy, the better... I'm not saying it's an anti-capitalist measure, but it's a challenge to it." (P24)	controlen; que cuanto más trabajo, mucho mejor; que cuanto más compras, mucho mejor... ¿no? No digo que sea una medida anticapitalista, pero sí un desafío.” (P24)
	"It would be quite a radical change from our parents. My father worked from the age of 13 to be able to have food and a house. And so they believe that leisure is not as important as work, and that you have to work hard to survive. So it would be a very radical change in those beliefs." (P9)	“Sería un cambio bastante radical respecto a nuestros padres. Mi padre estaba trabajando con 13 años, para poder tener comida y casa. Y eso les mete unas creencias de que el ocio no es tan importante como el trabajo, y que hay que esforzarse para poder tener lo mínimo para vivir. Entonces, sería un cambio muy radical en esas creencias.” (P9)
Potential for social change	"We could free up more time for ourselves. And just by freeing up that time, we would have the opportunity to think about other things, which we don't think about now, because we are stuck in the same old wheel... Maybe, as a society, we learn new values, right? Because, all of a sudden, there is a space for new values and new ways of living." (P7)	“Podríamos liberar más tiempo para nosotres. Y ya solo liberando ese tiempo, también tienes la oportunidad de pensar en otras cosas, que a lo mejor ahora no estás pensando, porque estás metide como en la rueda de siempre… A lo mejor, como sociedad, aprendemos valores nuevos, ¿no? Porque, de repente, hay un espacio para valores nuevos y formas de vida nuevas.” (P7)
